# Specific PP2A Catalytic Subunits Are a Prerequisite for Positive Growth Effects in Arabidopsis Co-Cultivated with *Azospirillum brasilense* and *Pseudomonas simiae*

**DOI:** 10.3390/plants10010066

**Published:** 2020-12-30

**Authors:** Irina O. Averkina, Ivan A. Paponov, Jose J. Sánchez-Serrano, Cathrine Lillo

**Affiliations:** 1 Department of Chemistry, Bioscience and Environmental Engineering University of Stavanger, 4036 Stavanger, Norway; irina.o.averkina@uis.no; 2 Norwegian Institute of Bioeconomy Research, Division of Food Production and Society, P.O. Box 115, NO-1431 Ås, Norway; ivpa@food.au.dk; 3 Departamento de Genética Molecular de Plantas, Centro Nacional de Biotecnología, 28049 Madrid, Spain; jjss@cnb.csic.es

**Keywords:** Arabidopsis, *Azospirillum brasilense*, plant growth-promoting bacteria, PGPR, PP2A, *Pseudomonas simiae*, protein phosphatase 2A

## Abstract

Plant growth-promoting rhizobacteria (PGPR) stimulate plant growth, but the underlying mechanism is poorly understood. In this study, we asked whether PROTEIN PHOSPHATASE 2A (PP2A), a regulatory molecular component of stress, growth, and developmental signaling networks in plants, contributes to the plant growth responses induced by the PGPR *Azospirillum brasilense* (wild type strain Sp245 and auxin deficient strain FAJ0009) and *Pseudomonas simiae* (WCS417r). The PGPR were co-cultivated with Arabidopsis wild type (WT) and PP2A (related) mutants. These plants had mutations in the PP2A catalytic subunits (*C*), and the PP2A activity-modulating genes *LEUCINE CARBOXYL METHYL TRANSFERASE 1* (*LCMT1*) and *PHOSPHOTYROSYL PHOSPHATASE ACTIVATOR* (*PTPA*). When exposed to the three PGPR, WT and all mutant Arabidopsis revealed the typical phenotype of PGPR-treated plants with shortened primary root and increased lateral root density. Fresh weight of plants generally increased when the seedlings were exposed to the bacteria strains, with the exception of catalytic subunit double mutant *c2c5*. The positive effect on root and shoot fresh weight was especially pronounced in Arabidopsis mutants with low PP2A activity. Comparison of different mutants indicated a significant role of the PP2A catalytic subunits C2 and C5 for a positive response to PGPR.

## 1. Introduction

Plant growth-promoting rhizobacteria (PGPR) positively influence plants by affecting hormone signaling, protecting plants against pathogens, and promoting the uptake of nutrients [1]. Such bacteria are expected to be important in developing better and more sustainable agricultural practices, but the underlying mechanisms and genes involved in the plant-bacteria communication are still debated. *Pseudomonas* species, which are among the most abundant bacteria in the microbiome of Arabidopsis and other plants [2,3] are candidates for improving agricultural practises. *Pseudomonas simiae* (formerly *Pseudomonas fluorescens*) has been characterized in detail, especially *P. simiae* WCS417r, and was found to stimulate biomass production, promote lateral root formation, promote auxin signaling, and trigger induced systemic resistance [3,4]. Genome wide association studies (GWAS) performed with Arabidopsis to identify plant genes important for the increased fresh weight and root growth related to *P. simiae* treatment, pointed to several interesting genes [5]. Among these genes was *PTPA* (*PHOSPHOTYROSYL PHOSPHATASE ACTIVATOR*), a PP2A (PROTEIN PHOSPHATASE 2A) activator that was putatively related to changes in lateral root formation (Supplementary Table S2 in [5]). Inspired by this study and our long-term interest in PP2A, a *PTPA* mutant and other mutants related to the PP2A complex were included in the study presented here.

*Azospirillum brasilense* is also well known for its ability to influence root architecture and increase growth in plants. This bacterium is already commercially applied to various crops [6]. Generally when plants are exposed to *A. brasilense*, lateral roots increase in number, length, and thickness while the main root is shortened (reviewed in Fibach-Paldi et al. 2011) [6]. These effects have been attributed to the auxin production in *A. brasilense*, and also the production of bacterial NO appeared crucial [6]. Other signaling compounds from *A. brasilense*, which are anticipated to influence plant growth, are cytokinins and gibberellins. These bacteria also ameliorate the response of Arabidopsis to drought by enhancement of ABA (abscisic acid) levels [7]. Furthermore, *A. brasilense* can improve nutrient uptake, including nitrogen uptake, in non-nodulating as well as nodulating plants [8], and has been suggested as a remedy to increase nitrogen use efficiency in important crops such as wheat [9]. Since bacterial plant-growth promoting effects are often, at least partly, caused by auxin produced by the PGPR, a well-characterized auxin synthesis mutant *A. brasilense* (FAJ0009) was included in our study. Use of both wild type *A. brasilense* (Sp245) and FAJ0009 for testing effects on Arabidopsis, should help in uncovering how PP2A could be involved in plant growth stimulated by bacteria. Global gene expression and growth parameters had already been investigated in Arabidopsis by Spaepen and collaborators and revealed valuable data indicating a more rapid and much stronger response for the auxin-producing *A. brasilense* strain than for FAJ0009 [10].

PP2A is responsible for a considerable part of the protein phosphatase activity in all eukaryotic cells. The complete PP2A complex consists of three subunits: a catalytic (C), a scaffolding (A), and a regulatory (B) subunit [11,12]. The carboxyl terminal end of the catalytic subunit becomes methylated by LCMT1 (LEUCINE CARBOXYL METHYL TRANSFERASE 1). Formation and optimal activity of the PP2A complex is promoted by PTPA and LCMT1 [13]. The activation process and physiological consequences exerted by these regulatory proteins are still poorly understood, but PP2A methylation is essential for coping with a wide range of stress conditions, including oxidative and salt stress [13,14]. Knockout of *LCMT1* gives predominantly, or exclusively, a non-methylated PP2A enzyme [14,15]. Absence of methylation leads to decreased PP2A activity and preferences for certain subunits to be included in the full PP2A complex [16]. Interestingly, Jin et al. (2016) [17] found that mutation of the yeast *PTPA* homolog (*RRD2*), *LCMT1* homolog (*PPM1*), or *PP2A* catalytic subunit (*PPH21*) permitted yeast to better tolerate expression of a bacterial type-III effector protein from a maize pathogen, indicating that PP2A and its regulators interfere with the bacterial effector protein. In the present work we included an Arabidopsis mutant that overexpressed the *PTPA* gene and thereby possessed higher PP2A activity than WT Arabidopsis. Furthermore, a *lcmt1* knockout mutant with lack of PP2A methylation and decreased PP2A activity was included.

The PP2A catalytic subunits fall into two subfamilies in all land plants tested [18]. Subgroup 1 has been found to be involved in stress and defence responses [19,20,21,22]. Subgroup 2 has been found to be involved in regulation of auxin fluxes through dephosphorylation of PIN proteins [23], in cell division [24], and involved in regulation at the receptor level for signaling from flagellin and elongation factor Tu [25]. Plants do not have an adaptive immune system like animals, but they respond to pathogen invasion by help of an innate immune system. Many reactions occur within minutes of the pathogen attack, indicating post-translational events. Recent research points to an overlap between innate immunity and the plant mechanisms for responding to symbiotic and growth-promoting microorganisms [26]. Silencing of subfamily 1 in *Nicotiana benthamiana* resulted in constitutively expressed pathogen-related (PR) genes, and the plants developed localized cell death in stems and leaves [19]. The work with *N. benthamiana* indicated that PP2A catalytic subfamily 1 members acted as negative regulators of plant defence responses, possibly by de-sensitizing protein phosphorylation cascades. For tomato, He and collaborators [19] showed that a PP2A catalytic subunit, also belonging to subfamily 1, was rapidly induced when exposed to a *Pseudomonas syringae* strain. For Arabidopsis, the TAIR database indicates that treatment with *Pseudomonas syringae* or *Phytophthora infestans* led to higher expression of *C1* and *C5*, respectively (both subgroup 1) [27]. Also, a PP2A catalytic subunit of the subgroup 2 was reported to be involved in the response to *P. syringae*. Indeed, Arabidopsis plants with knocked out *PP2A-C4* were more resistant towards *P. syringae* than WT Arabidopsis [25]. Complete knockout of all three subunits belonging to subgroup 1 cripples the Arabidopsis seedlings [23], but double mutants grow relatively well, and we decided to test how plant growth-promoting bacteria influence various double mutants and the *c4* single mutant.

To explore the involvement of PP2A in PGPR-plant interactions we chose three frequently studied and well-characterized bacterial strains as model organisms. These strains were *A. brasilense* Sp245 and FAJ0009, and *P. simiae* WCS417r. Our results revealed differences between Arabidopsis WT and *pp2a* mutants in their response to the PGPR, and that PGPR ameliorated growth in mutants with low PP2A activity with the exception of the *c2c5* double mutant.

## 2. Results

### 2.1. PP2A Activity in Arabidopsis WT and Selected Mutants

**Figure 1:**
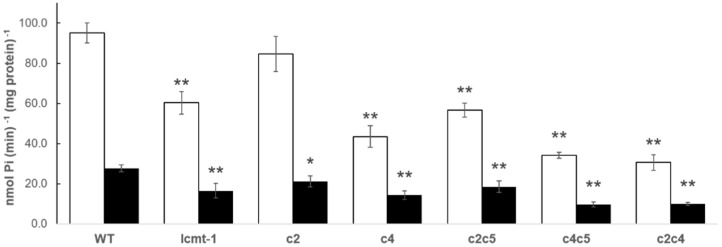
PP2A activity in selected Arabidopsis mutants. Activity was assayed separately in roots (white bars) and shoots (black bars) from 10-d-old seedlings grown on one-half MS medium. SE is given (*n* = 3, three biologically independent experiments), two asterisks indicate that values are significantly different from WT according to Student’s *t*-test with *p* < 0.05, and for one asterisk with *p* < 0.1.

PP2A activity was tested in both roots and shoots of WT, *lcmt1*, two single (*c2* and *c4*) and three double (*c2c5*, *c4c5* and *c2c4*) mutants of the PP2A catalytic subunits (Figure 1). PP2A activity in roots was always much higher than in shoots (on a per mg protein basis). The low activity of these mutants had been found also in extracts from whole seedlings (S1). Mutants of *ptpa* had previously been studied in detail [13,14] and the *ptpa_ox_* was revealed to be a high activity mutant with 180% PP2A activity compared with WT [14]. The *lcmt1*, *ptpa_ox_*, *c4* single mutant, and the three double mutants *c2c5*, *c4c5*, and *c2c4* were included in further experiments to characterize interactions between PGPR and PP2A.

### 2.2. PP2A Activity in Arabidopsis WT Treated with PGPR

**Figure 2:**
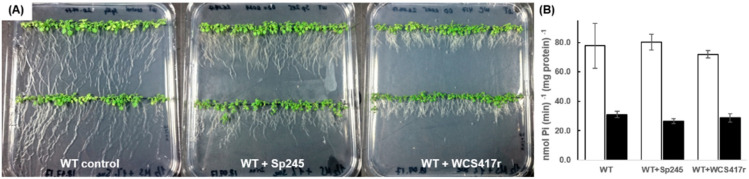
Visual phenotype and PP2A activity of Arabidopsis WT not treated or treated with PGPR. (**A**) Control plants were grown without bacteria (left plate) or co-cultivated with *A. brasilense* Sp245 (middle plate), or with *P. simiae* WCS417r (right plate) for 14 days. (**B**) Activity was assayed separately in roots (white bars) and shoots (black bars). Experiments were repeated three times. Values for plants treated with bacteria were not significantly different from non-treated plants at *p* < 0.1, SE is given.

To check if PP2A activity was influenced by treatment with PGPR, PP2A activity in roots and shoots of Arabidopsis seedlings exposed to *P. simiae* or *A. brasilense* for two weeks was compared with non-treated control plants. Despite the pronounced phenotypic response observed for plants grown with bacteria, total in vitro PP2A activity did not deviate significantly from the control seedlings grown in the absence of bacteria (Figure 2).

### 2.3. Phenotypic Response of Arabidopsis WT and Mutants to P. simiae WCS417r

**Figure 3:**
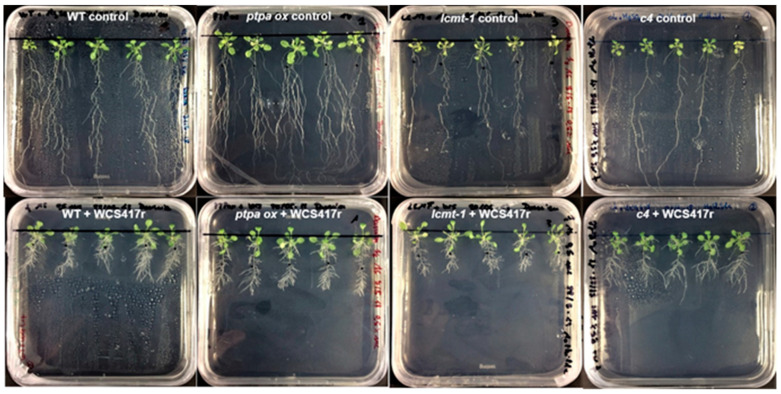
Visual phenotype of Arabidopsis WT and mutants treated with *P. simiae* WCS417r. Seedlings of Arabidopsis WT and mutants (*ptpa_ox_*, *lcmt1*, *c4*) cultivated without (upper row) and with *P. simiae* WCS417r (lower row) for two weeks.

**Figure 4:**
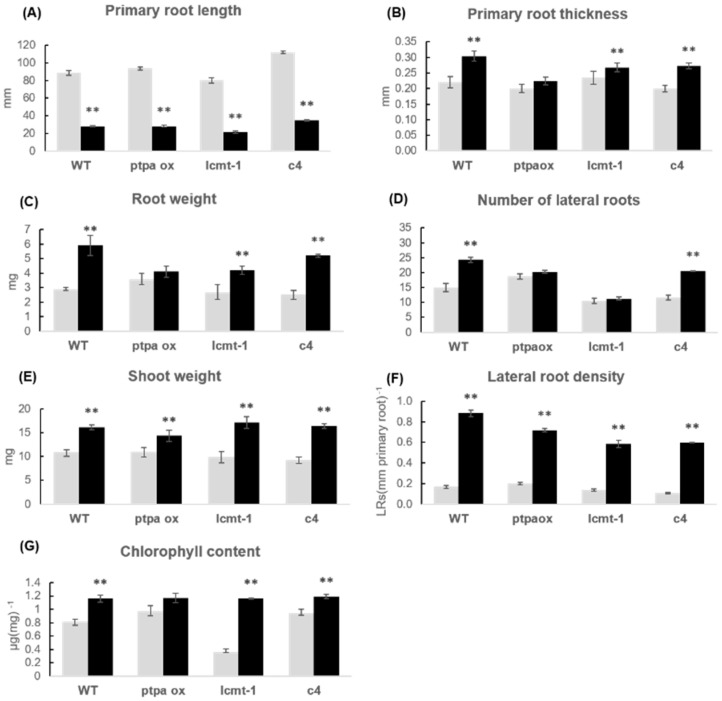
Growth parameters of WT and mutants treated with *P. simiae* WCS417r. Seedlings of Arabidopsis WT, *ptpaox*, *lcmt1*, and *c4* were cultivated without (grey bars) and with (black bars) *P. simiae* WCS417r for two weeks. (**A**) primary root length; (**B**) primary root thickness; (**C**) root fresh weight; (**D**) number of lateral roots; (**E**) shoot fresh weight; (**F**) lateral root density; and (**G**) chlorophyll content. For root length, primary root thickness, number of lateral roots, and root density, data are means ± SE of 30 plants (*n* = 30). For fresh weight and chlorophyll content, the plants in each plate were pooled for measurements, and data given is the average of six independent experiments, *n* = 6, ±SE is given. The root and shoot weights (**C**,**E**) represent single plant averages. The chlorophyll values (**G**) represent µg chlorophyll per mg leaf tissue. According to Student’s *t*-test and *p*-value < 0.05, columns marked with two asterisks are significantly different from the control without bacteria.

**Figure 5:**
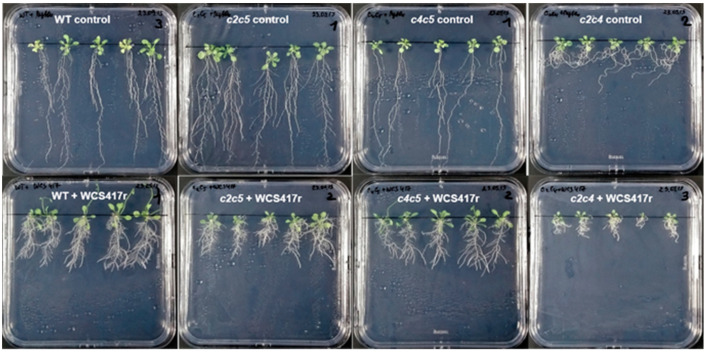
Visual phenotype of Arabidopsis WT and double mutants treated with *P. simiae* WCS417r. Seedlings of Arabidopsis WT and double mutants *c2c5*, *c4c5*, and *c2c4* were cultivated without (upper row) and with *P. simiae* WCS417r (lower row) for two weeks.

**Figure 6:**
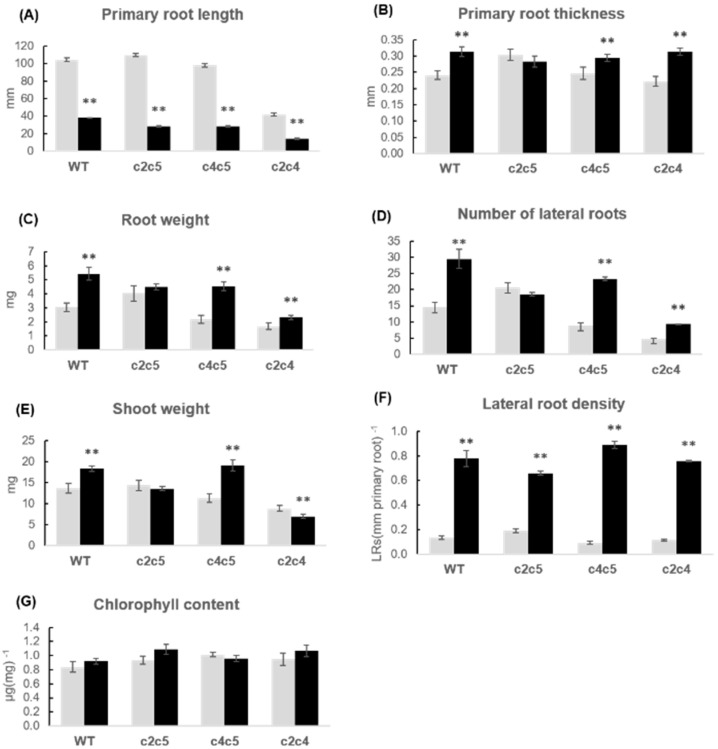
Growth parameters of WT and double mutants treated with *P. simiae* WCS417r. Seedlings of Arabidopsis WT and double mutants *c2c5*, *c4c5*, *c2c4* were cultivated without (grey bars) and with (black bars) *P. simiae* WCS417r for two weeks. (**A**) primary root length; (**B**) primary root thickness; (**C**) root fresh weight; (**D**) number of lateral roots; (**E**) shoot fresh weight; (**F**) lateral root density; and (**G**) chlorophyll content. For root length, primary root thickness, number of lateral roots, and root density, data are means ± SE of 30 plants (*n* = 30). For fresh weight and chlorophyll content, the plants in each plate were pooled for measurements, and data given is the average of six independent experiments, *n* = 6, ±SE is given. The root and shoot weights (**C**) and (**E**) represent single plant averages. The chlorophyll values (**G**) represent µg chlorophyll per mg leaf tissue. According to Student’s *t*-test and *p*-value < 0.05, columns marked with two asterisks are significantly different from the control without bacteria.

The cultivation of Arabidopsis seedlings with *P. simiae* WC417r was performed in two series. These two series are presented in separate diagrams with their respective WT reference. Arabidopsis lines including WT, *ptpa_ox_, lcmt1,* and *c4* were first studied (Figure 3 and Figure 4), and the second series included WT and the three double mutants *c2c5*, *c4c5*, and *c2c4* (Figure 5 and Figure 6). Irrespective of the genotype, the root architecture of all seedlings was strikingly influenced by *P. simiae*, with a shorter primary root (Figure 3, Figure 4A, Figure 5, and Figure 6A). Averaged across all genotypes, primary root length was 31 ± 1% (spreading from 26–36%) of non-treated seedlings. Primary root thickness and root weight significantly increased for all genotypes, except for *ptpa_ox_* and *c2c5* which were not significantly influenced by *P. simiae* (Figure 4B,C, and Figure 6B,C). Number of lateral roots clearly increased in WT, *c4*, *c4c5*, and *c2c4*, but did not change significantly in *ptpa_ox_*, *lcmt1*, and *c2c5* in response to *P. simiae* (Figure 4D and Figure 6D). However, all genotypes clearly showed increased lateral root density (number of lateral roots per mm primary root) (Figure 4F and Figure 6F). Shoot weight generally increased in response to *P. simiae*, but not for double mutants with knocked out *c2*—i.e., *c2c5* and *c2c4* (Figure 4E and Figure 6E). Chlorophyll content was unchanged or increased in plants treated with *P. simiae*, strikingly so for the *lcmt1* mutant (Figure 4G and Figure 6G). *Lcmt1* originally had low chlorophyll content compared with WT, but after inoculation with *P. simiae* reached WT values (Figure 4G).

Effects of PGPR (*P. simiae*) were tested also with some *pp2a-b’* mutants (*b’alpha, b’beta*, *b’gamma, b’zeta, b’theta)*. These B’ subunits had previously been implicated in the response to bacterial pathogens [17,25,28]. The mutants were not studied in detail, but the experiments showed that they all responded to bacteria treatment with shortened primary root and increased lateral root density. The response of *b’alpha* and *b’theta* were different from WT, with significant fewer lateral roots and lower lateral root density (Figures S2 and S3). Interestingly, *b’theta* had previously been shown to interact with C2 and C5 in vivo, and the current results are in agreement with the earlier suggestion [29] that C2 and C5 are part of PP2A complexes containing B’theta.

### 2.4. Phenotypic Response of Different Arabidopsis WT and Mutants Treated with Azospirillum brasilense Sp245 and FAJ0009

**Figure 7:**
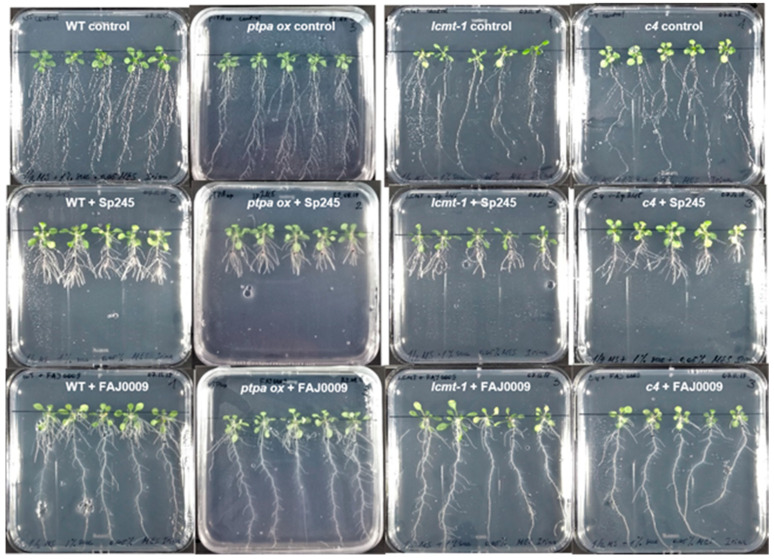
Visual phenotype of Arabidopsis WT and mutants treated with *A. brasilense*. Seedlings of Arabidopsis WT and mutants *ptpa_ox_*, *lcmt1*, *c4* were cultivated without bacteria (upper row) and with *A. brasilense* Sp245 (middle row) and *A. brasilense* FAJ0009 (lower row) for two weeks.

**Figure 8:**
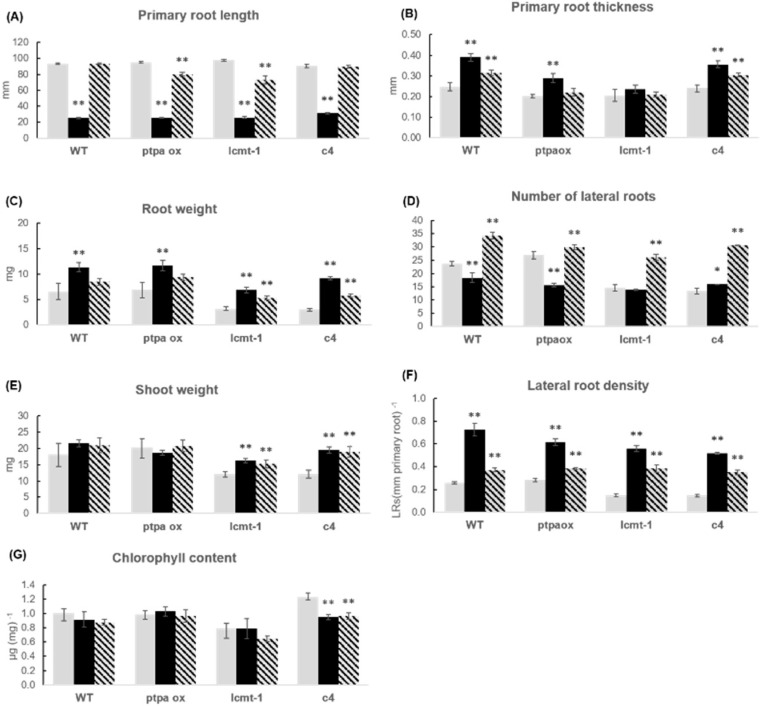
Growth parameters of WT and mutants influenced by *A. brasilense*. Growth parameters of WT and mutants *ptpa_ox_*, *lcmt1*, *c4* cultivated without bacteria (grey bars), with *A. brasilense* Sp245 (black bars) and with *A. brasilense* FAJ0009 (hatched bars) for two weeks. (**A**) primary root length; (**B**) primary root thickness; (**C**) root fresh weight; (**D**) number of lateral roots; (**E**) shoot fresh weight; (**F**) lateral root density; and (**G**) chlorophyll content. For root length, primary root thickness, number of lateral roots, and root density, data are means ± SE of 30 plants (*n* = 30). For fresh weight and chlorophyll content, the plants in each plate were pooled for measurements, and data given is the average of six independent experiments, *n* = 6, ±SE is given. The root and shoot weights (**C**,**E**) represent single plant averages. The chlorophyll values (**G**) represent µg chlorophyll per mg leaf tissue. According to Student’s *t*-test and *p*-value < 0.05, columns marked with two asterisks are significantly different from the control without bacteria, and for one asterisk with *p* < 0.1.

**Figure 9:**
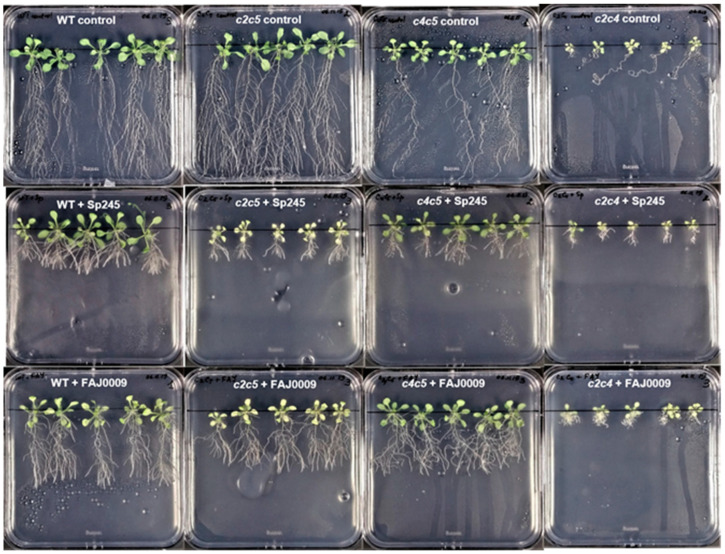
Visual phenotype of Arabidopsis WT and double mutants treated with *A. brasilense*. Seedlings of Arabidopsis WT and double mutants *c2c5*, *c45*, *c2c4* were cultivated without bacteria (upper row) and with *A. brasilense* Sp245 (middle row) and with *A. brasilense* FAJ0009 (lower row) for two weeks.

**Figure 10:**
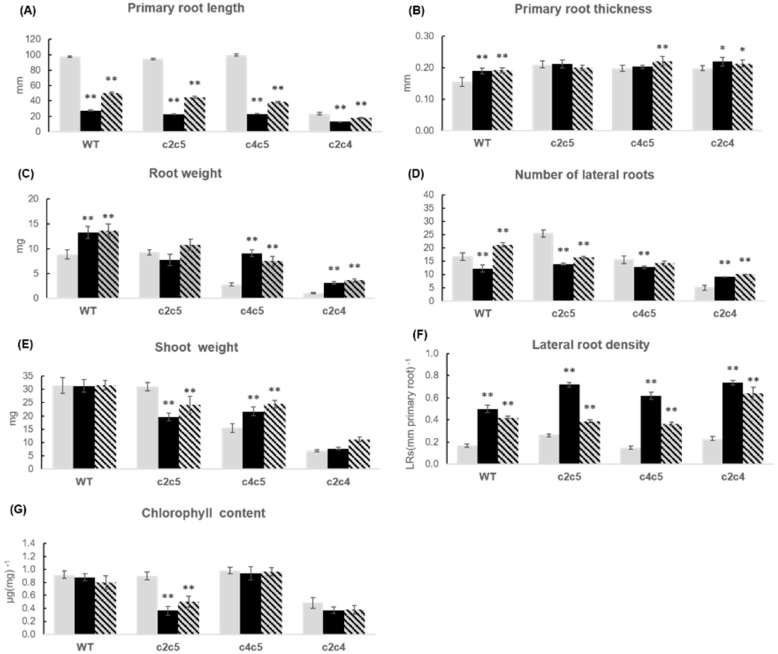
Growth parameters of WT and double mutants treated with *A. brasilense*. Growth parameters of WT and double mutants *c2c5*, *c4c5*, *c2c4* cultivated without bacteria (white bars), with *A. brasilense* Sp245 (black bars) and with *A. brasilense* FAJ0009 (hatched bars) for two weeks. (**A**) primary root length; (**B**) primary root thickness; (**C**) root fresh weight; (**D**) number of lateral roots; (**E**) shoot fresh weight; (**F**) lateral root density; and (**G**) chlorophyll content. For root length, primary root thickness, number of lateral roots, and root density, data are means ± SE of 30 plants (*n* = 30). For fresh weight and chlorophyll content, the plants in each plate were pooled for measurements, and data given is the average of six independent experiments, *n* = 6, ±SE is given. The root and shoot weights (**C**) and (**E**) represent single plant averages. The chlorophyll values (**G**) represent µg chlorophyll per mg leaf tissue. According to Student’s *t*-test and *p*-value < 0.05, columns marked with two asterisks are significantly different from the control without bacteria, and for one asterisk with *p* < 0.1.

Two different strains of *A. brasilense* were used for inoculation: the auxin producing wild type strain Sp245 and an auxin synthesis mutant, FAJ0009. The FAJ0009 strain is mutated in the IpdC gene resulting in an auxin production reduced by more than 90% [30]. As for *P. simiae* the experiments were divided into two series: WT, *ptpa_ox_, lcmt1* and *c4* (Figure 7 and Figure 8), and WT, *c2c5*, *c4c5*, and *c2c4* (Figure 9 and Figure 10). WT and all mutants were influenced by *A. brasilense*, both Sp245 and FAJ0009 (Figure 7, Figure 8, Figure 9 and Figure 10). Irrespective of the genotype, the root architecture of all seedlings was strikingly influenced by the Sp245 strain (Figure 7, Figure 8A, Figure 9, and Figure 10A). Across all the experiments, the average primary root length for Sp245 treated plants was 31 ± 4% of non-treated (spread from 23 to 53%), that is a similar effect as observed for *P. simiae*. A less pronounced effect was seen for plants treated with FAJ0009 giving an average root length of 71 ± 8.0% compared with non-treated plants (spread from 34–100%). In the first series there was no significant root shortening on WT and *c4* mutant, but in the second series root shortening by FAJ0009 was significant for all genotypes, including WT. Apparently, small variations in experimental conditions may have had a large influence, that however, did not change the overall trends. An increase in primary root thickness or root weight was observed for all genotypes, at least for one of the two bacteria Sp245 or FAJ0009, with the striking exception of the *c2c5* mutant which showed no effects (Figure 8B,C and Figure 10B,C).

Such a lack of response with *c2c5* had also been observed for *P. simiae* (Figure 6B,C). Number of lateral roots decreased for *c2c4*, but other genotypes showed variable responses with Sp245 and FAJ0009 (Figure 8D and Figure 10D). Lateral root density increased in all genotypes in response to Sp245 as well as FAJ0009 treatment. The response to Sp245 was, however, always stronger than to FAJ009, which induced a response in-between non-treated and Sp245-treated (Figure 8F and Figure 10F). Shoot fresh weight was usually higher or unchanged in seedlings treated with Sp245 or FAJ0009 compared with non-treated plants. However, for the *c2c5* mutant a clear decrease in shoot fresh weight was observed for treatment with both Sp245 and FAJ0009 (Figure 10E). Chlorophyll content was not positively influenced by Sp245 or FAJ0009. Notably, chlorophyll content in the *c2c5* mutant responded with a significant and strong decrease after co-cultivation with both strains of *Azospirillum* (Figure 10G).

## 3. Discussion

When Arabidopsis or other plants are exposed to PGPR bacteria, general effects on root architecture repeatedly observed in different laboratories are shortened primary root and increased lateral root density. Such a change in root architecture was observed for WT, *lcmt1*, *ptpa_ox_*, and all *pp2a* mutants tested, showing that these basic responses occurred in plants with both high and low PP2A activity and irrespective of the methylation state of the PP2A catalytic subunits (A and F in Figure 4, Figure 6, Figure 8, and Figure 10). All three bacteria strains, *P. simiae*, and *A. brasilense* Sp245 and FAJ0009, induced short primary roots and increased lateral root density, but the response to FAJ0009 was much weaker. Although a response was observed in all Arabidopsis lines tested, a closer look revealed different growth responses among genotypes. Changes in root parameters for the *ptpa_ox_* mutant were very similar to WT when exposed to *A. brasilense* Sp245 or FAJ0009, but different from WT when exposed to *P. simiae* WCS417r. When treated with *P. simiae*, primary root thickness, root weight, and number of lateral roots, all increased for WT Arabidopsis, but none of these root parameters changed significantly for the *ptpa_ox_* mutant (Figure 4 and Figure 8). According to the GWAS (genome-wide association studies) by Wintermans et al. (2016) [5], the PTPA locus was a candidate to induce deviations regarding number of lateral roots when treated with *P. simiae*, however not a candidate to induce deviations regarding shoot fresh weight or primary root length. Indeed, our experiments with the *ptpa_ox_* mutant showed that only the response of number of lateral roots was different from WT. Since we worked with an overexpressor and observed a negative response on the number of lateral roots, the PTPA locus with a positive response in the GWAS could be a locus linked with decreased PTPA expression. This hypothesis would need further investigation and requires a suitable *ptpa* mutant with decreased/knocked-down PTPA expression that may allow to establish the importance of the locus for positive responsiveness to PGPR.

In the *lcmt1* mutant, the catalytic subunits are mainly in the non-methylated form, and this leads to decreased PP2A activity (Figure 2 and [13]). The physiological function of PP2A methylation is still enigmatic in plants, but apparently lack of methylation did not hinder positive effects from bacteria. Both root and shoot weight of *lcmt1* were positively influenced by the bacteria strains tested, and *P simiae* WCS417 also ameliorated the low chlorophyll content in *lcmt1* (Figure 4G).

Auxin, or compounds stimulating auxin signaling in plants, clearly can promote plant root growth, as has frequently been reported for various *Pseudomonas* [3,31,32] and *Azospirillum* strains [10,30]. It has previously been shown that incubation with *P. simiae* WCS417r induced genes in Arabidopsis with a strong auxin signature, and these genes stimulated the changes in root architecture and promoted plant growth [3,32]. When Arabidopsis was co-cultivated with *A. brasilense Sp245* or the auxin synthesis mutant FAJ0009 for two weeks, we observed a clear, but much weaker, effect on root architecture for FAJ0009. This confirmed that auxin produced by *A. brasilense* strongly influenced root architecture but was most likely not the only bacterial factor influencing plant growth (Figure 8 and Figure 10).

Striking phenotypic results were obtained for the *c2c5* mutant. In contrast with the other genotypes, the *c2c5* mutant was not significant positively influenced by any of the three bacteria strains regarding primary root thickness, root weight, number of lateral roots, shoot weight, or chlorophyll content. The C2 and C5 subunits, together with C1, make up the subgroup 1 of PP2A catalytic subunits in Arabidopsis. Apparently, when both C2 and C5 were knocked out, their functions could not be fully replaced by the C1 subunit, although the three subunits may have some overlapping functions. Catalytic subgroup 1 members are suppressors of defence responses in higher plants, and C2 and C5 are therefore candidates to be involved in defence signaling processes in Arabidopsis induced by bacteria [19,28]. Lack of these catalytic subunits could possibly hamper the silencing of defence genes necessary for obtaining a balanced trade-off between growth and defence that could promote growth by PGPR. Another possibility would be that lack of C2 and C5 would impair auxin signaling; however, only group 2 catalytic subunits (C3, C4) have been shown to be important for auxin signaling. PIN (PIN FORMED) proteins are auxin transporters which are often localised in the membrane at the polar ends of cells in the direction of polar auxin transport [33]. The PP2A dephosphorylation activity is important for counteracting PID (PINOID)-mediated phosphorylation of PIN proteins. This dephosphorylation determines proper PIN localisation and facilitates polar auxin efflux from cells. PP2A catalytic subunits C3 and C4 were previously shown to be important for dephosphorylation of PINs, but single mutants of these subunits showed wild type phenotype. Only a double mutant *c3c4* showed mislocalization of PIN proteins, indicating the contribution of these subunits and their redundant roles in controlling of PIN polarity [23]. Since PP2A affects auxin signaling probably through the polar auxin transport and subgroup 2 catalytic subunits, effects of silencing subgroup 1 catalytic subunits are more likely to be related to other processes, like regulation of defence or ABA signaling. PP2A catalytic subunits C2 and C5 are necessary for optimal root growth under stress conditions such as high salt concentrations and exposure to ABA [20,22]. Expression analysis using the C2 promoter linked with GUS had shown that C2 expression correlated with root development and suggested a role for C2 in lateral root growth. Under standard growth conditions, roots of the *c2* mutant were not distinguishable from WT roots, but when exposed to ABA the *c2* mutant showed a more severe inhibition of root growth than did WT. Furthermore, a C2 overexpressor was less influenced by ABA than WT [20]. A *c5* mutant studied by Hu et al. (2017) was hypersensitive to various salts and developed shorter roots and produced smaller leaves under stress than WT [22]. The work also showed that C5 was involved in salt tolerance most likely through interactions with chloride channel proteins in the vacuole membrane. Amelioration of growth by *A. brasilense* Sp245 during drought was shown to involve ABA production [7,34]. The effects observed in the *c2c5* mutant in the present study could be explained by lack of these subunits, which otherwise would mediate ABA and stress signaling. This would fit with previous investigations that *A. brasilense* Sp245 did produce ABA and that co-cultivation with plants induced a two-fold higher level of endogenous ABA in Arabidopsis. ABA is associated with abiotic stress like high salt concentrations and drought and is also involved in biotic stress. ABA thus functions at the crossroad of abiotic and biotic stress responses [35]. The pathogenic *Pseudomans syringae* also induced a response in Arabidopsis which clearly involved ABA [36]. If ABA signaling could explain the effects observed when *c2c5* was co-cultivated with *P. simiae* WCS417r remains to be investigated because the response triggered by *P. simiae* was clearly different from *P. syringae* [32]. Both C2 and C5 overexpressors improved root growth under various abiotic stress treatments [20,22], and it would be interesting to explore if overexpression of these genes could enhance the positive effects from PGPR beyond that observed in WT plants.

## 4. Materials and Methods

### 4.1. Plant Material

*Arabidopsis thaliana* Columbia ecotype, *lcmt1* knockout line (insertion in At1g02100 exon four, SALK_079466 [37], *ptpa* overexpressor GABI_606E07 (insertion in promoter) [38] were provided by the European Arabidopsis Stock Centre in Nottingham UK, and previously characterized [14]. PP2A catalytic subunit single and double mutants derived from SALK and SAIL lines were also described previously [14,23,25,39].

### 4.2. Bacterial Strains

*Azospirillum brasilense* Sp245 wild-type strain [10] and its ipdC-knockout mutant FAJ000 (Sp245 *ipdC*:Tn5) impaired in auxin biosynthesis capacity because of knockout mutation in the key gene for auxin biosynthesis Indole-3-pyruvate decarboxylase (ipdC) [30,40], were kindly provided by Laurent Legendre (Saint-Étienne, France) and Claire Prigent-Combaret (CNRS, Lyon, France). *Pseudomonas simiae* (formerly *Pseudomonas fluorescens*) WCS417r is the rifampicin-resistant mutant of the biocontrol strain *Pseudomonas simiae* WCS417 originally isolated from the rhizosphere of wheat grown in Brazil [41], was kindly provided by Corné M.J. Pieterse and I.A. Hans van Pelt, Department of Biology, Utrecht University, Utrecht, the Netherlands.

### 4.3. Plant Growth Conditions for Bacteria Treatment

Surface sterilized seeds of Arabidopsis were sown in square Petri dishes (120 × 120 mm) containing half-strength Murashige and Skoog (MS) medium [42] with 1% (*w/v*) sucrose, pH 5.8, and 0.8% (*w/v*) agar (VWR International, Milano, Italy). The plates were placed in the dark at 4 °C for 2 days for stratification prior to cultivation vertically at a 16 h photoperiod for 5 days prior to transfer to new plates (five plants per plate) with bacteria-inoculated one-half MS medium.

### 4.4. Plant Growth Medium for Pseudomonas Treatment

Half-strength Murashige and Skoog and pH adjusted to 5.8. Sucrose (0.5%) and agar (0.8%) were added. After autoclaving the medium was poured into 12 × 12 cm Petri dishes.

### 4.5. Inoculating with Pseudomonas WCS 417r

*Pseudomonas* from glycerol stocks was streaked onto a Petri dish with King agar B supplemented with 50 mg L^−1^ rifampicin and incubated overnight at 28 °C. The grown bacterial culture was loosened in 10 mL of 10 mM MgSO_4_ [41]. The bacterial suspension was pipetted into a 15 mL Falcon tube and centrifuged at 3900× *g* for 5 min, pelleted bacteria were washed once in 10 mM MgSO_4_, then resuspended in 10 mM MgSO_4_ to obtain OD_600_ = 0.005 or 10^5^ cells/mL [43]. Bacterial suspension, 500 µL, was spread evenly onto each Petri dish before transferring the plants.

### 4.6. Plant Growth Medium for Azospirillum Treatment

Half-strength Murashige and Skoog was prepared with 2.56 mM Mes buffer and pH adjusted to 6.0. Sucrose (1%) and agar (0.8%) were added. After autoclaving the medium was poured into 12 × 12 cm Petri dishes.

### 4.7. Inoculating with Azospirillum spp. (Sp245 and FAJ0009)

*Azospirillum* Sp245 and FAJ0009 from glycerol stocks were streaked and grown on Luria–Bertani (LB) solid medium supplemented with 2.5 mM CaCl_2_, 2.5 mM MgSO_4_, and 50 mg L^−1^ kanamycin (only for FAJ0009) for 48 h at 37 °C. The colonies were used to produce an overnight culture in 5 mL of supplemented LB broth and incubated at 37 °C with shaking at 180 rpm overnight. The overnight culture (0.1 mL) was subcultured in 50 mL of supplemented LB broth and incubated at 37 °C under shaking at 180 rpm overnight. On the following day, the bacterial suspension was pipetted into 50 mL Falcon tubes and centrifuged at 3900× *g* for 5 min at 16 °C, the supernatant was removed, and the cells were re-suspended in 10 mL of 10 mM MgSO_4_, washed once with 0.85% NaCl and re-suspended in fresh 10 mM MgSO_4_ to obtain OD_600_ = 1 or 5 × 10^8^ cells/mL [10]. One volume of this suspension was mixed with 9 volumes of the warm (45 °C) plant growth medium (giving OD_600_ = 0.1). Ten mL of the mixture was poured onto 30 mL of already solidified plant growth medium and allowed to solidify.

### 4.8. Chlorophyll Assay

Five shoots from each plate were weighted and thoroughly ground with 2 mL of 95% ethanol in a mortar. The obtained suspension was transferred to a 2 mL collection tube and centrifuged for 1 min at 21,000× *g*. Prior to measuring absorbance at 654 nm, 300 µL of the supernatant was diluted with 1.2 mL of 95% ethanol. The chlorophyll content was calculated as A_654_ × 1000/39.8 × extraction volume (mL)/weight of fresh tissue (mg) × dilution factor of 5 = µg chlorophyll per mg leave tissue [44].

### 4.9. PP2A Assay

The Ser/Thr Phosphatase assay system from Promega (Promega 2009, Madison, WI, USA) was used as previously described in [39]. Frozen tissue, 100 mg, was homogenized and prepared for assay with 0.1 mM phosphorylated peptide (RR(pT)VA) as substrate. Phosphate released was measured, and assays with and without 5 nM okadaic acid were compared to calculate the PP2A activity [39]. For total phosphatase activity okadaic acid was omitted. After 15 min incubation, absorbance was read at 630 nm and converted into corresponding amounts of phosphate using a trend line equation of the standard curve (Promega. Technical Bulletin. Serine/Threonine Phosphatase Assay System. Instructions for use of product V2460, Madison, WI, USA). PP2A activity was calculated as the difference of phosphate released with and without okadaic acid [39,45].

## 5. Conclusions

Arabidopsis mutants with high or low PP2A activity due to mutations in PP2A catalytic and regulatory proteins all responded to *Pseudomonas simiae* WCS417r and *Azospirillum brasilense* Sp245 and FAJ0009 with a change in root architecture giving a shortened primary root and higher density of lateral roots. Additionally, most mutants responded to the PGPR by an increase in fresh weight, but a *pp2a* subgroup 1 double mutant with knocked out catalytic subunits *C2* and *C5* did not show increased fresh weight, indicating that these catalytic subunits play important roles for the ability to enhance growth induced by PGPR.

## Data Availability

Data used is within the article or Supplementary Material.

## Supplementary Materials

The following are available online at https://www.mdpi.com/2223-7747/10/1/66/s1, Figure S1: PP2A activity in Arabidopsis WT single (*c1*, *c2*, *c3*, *c4*, *c5*) and double (*c2c5*, *c4c5*, *c2c4*) mutants of PP2A catalytic subunits; Figure S2: Visual phenotype of Arabidopsis WT, *c2* and various *b’*-mutants treated with *P. simiae* WCS417r; Figure S3: Growth parameters of WT, *c2* and various *b’*-mutants treated with *P. simiae* WCS417r.
