# Supplementary material for: Specific PP2A Catalytic Subunits Are a Prerequisite for Positive Growth Effects in Arabidopsis Co-Cultivated with Azospirillum brasilense and Pseudomonas simiae
Source: Plants (Basel). 2020 Dec 30;10(1):66. doi: 10.3390/plants10010066 (PMC7823443; doi:10.3390/plants10010066)
Supplement: Supplementary file 1 [file plants-10-00066-s001.pdf]

Supporting information

Figure S1. PP2A activity in Arabidopsis WT single (*c1, c2, c3, c4, c5*) and double (*c2c5, c4c5, c2c4*) mutants of PP2A catalytic subunits.

Figure S2. Scatter plot of changes in Arabidopsis fresh weight caused by *P. simiae* WCS417r and related to PP2A activity ascribed to the different Arabidopsis mutants.

Figure S3. Scatter plot of changes in Arabidopsis fresh weight caused by *A. brasilense* and related to PP2A activity ascribed to the different Arabidopsis mutants.

Figure S4. Visual phenotype of Arabidopsis WT, *c2* and various *b'*-mutants treated with *P. simiae* WCS417r.

Figure S5. Growth parameters of WT, *c2* and various *b'*-mutants treated with *P. simiae* WCS417r

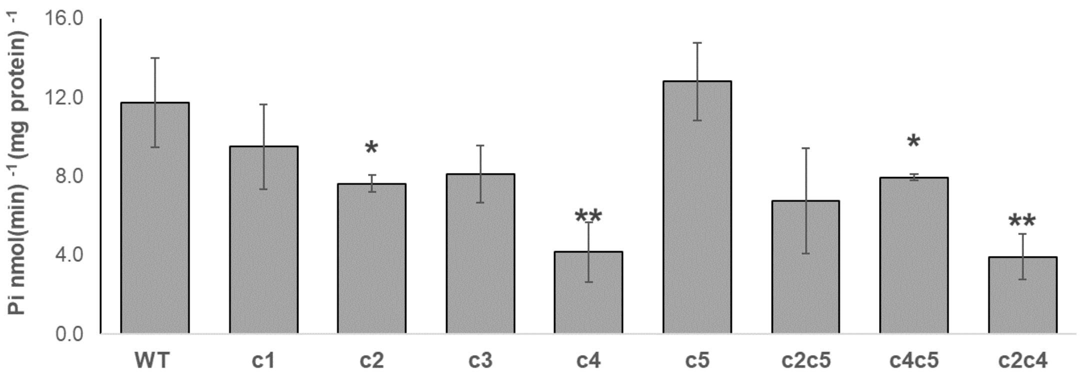

Figure S1. PP2A activity in Arabidopsis WT, single (*c1, c2, c3, c4, c5*) and double (*c2c5, c4c5, c2c4*) mutants of PP2A catalytic subunits.

Activity was assayed in the whole 10-d-old seedlings grown on ½ MS medium. SE is given (n=3, from three independent experiments), two asterisks indicate that values are significantly different from WT according to student's t-test at $p < 0.05$, and for one asterisk at $p < 0.1$.

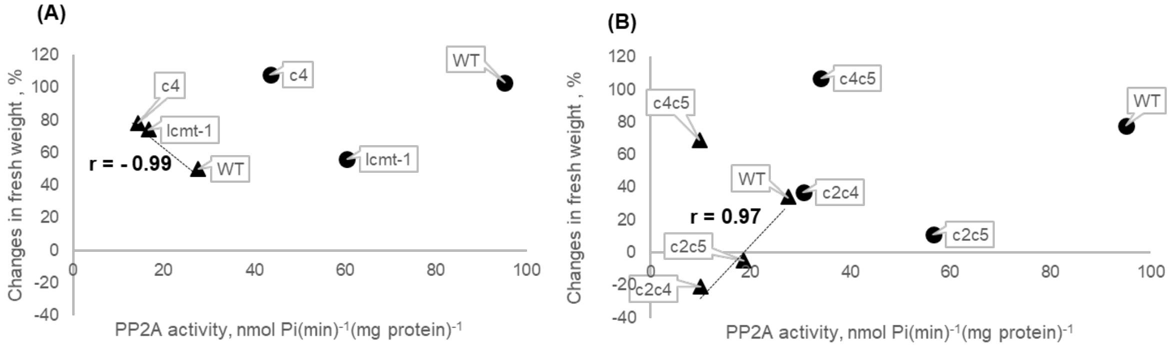

Figure S2. **Scatter plot of changes in Arabidopsis fresh weight caused by *P. simiae* WCS417r and related to PP2A activity ascribed to the different Arabidopsis mutants.**

Fresh weight changes in roots (circles) and shoots (triangles). Data are from Fig. 1, 4 and 6. (A) and (B) Only Pearson's correlation coefficient $|r| > 0.7$ was considered as strong, (B) *c4c5* was an outlier and not used in the calculation.

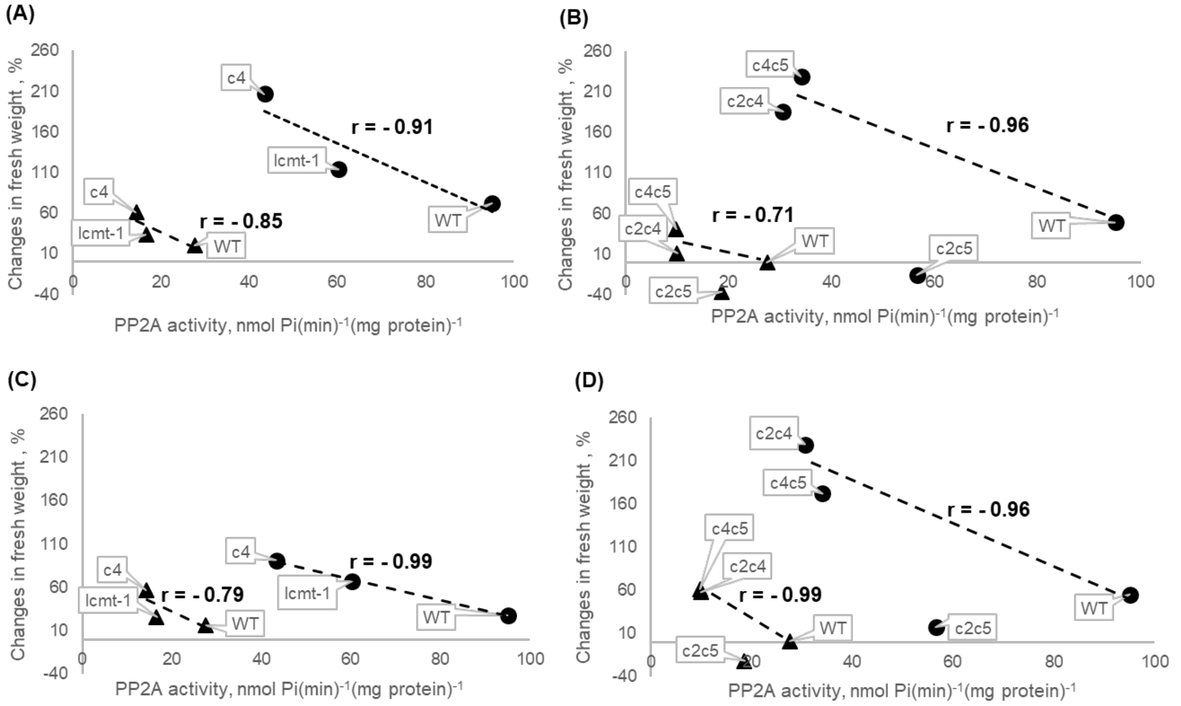

Figure S3. **Scatter plot of changes in Arabidopsis fresh weight caused by *A. brasilense* and related to PP2A activity ascribed to the different Arabidopsis mutants.**

Fresh weight changes in roots (circles) and shoots (triangles). Data are from Fig. 1, 9 and 11. (A) and (B) *A. brasilense* Sp245. (C) and (D) *A. brasilense* FAJ0009. Only Pearson's correlation coefficient $|r| > 0.7$ was considered as strong, (B) c2c5 and (D) *c2c5* were outliers and not used in the calculations.

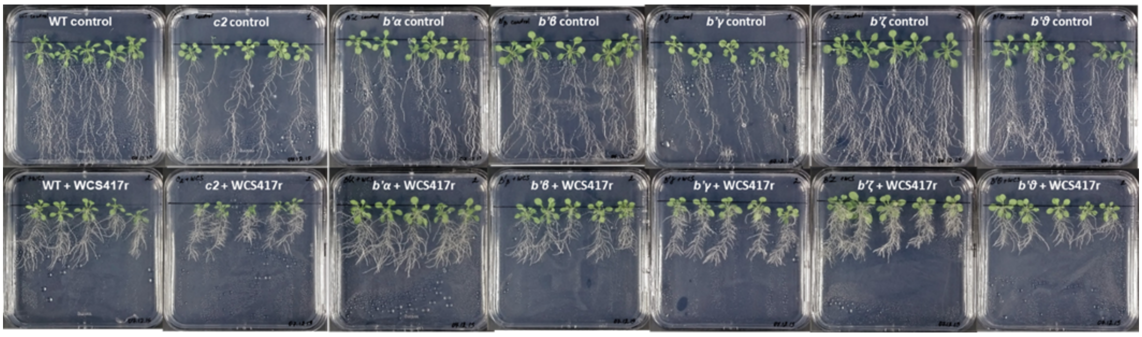

Figure S4. Visual phenotype of Arabidopsis WT, *c2* and various *b'*-mutants treated with *P. simiae* WCS417r. Seedlings of Arabidopsis WT and mutants (*c2*, *b*' alpha, beta, gamma, zeta, theta) cultivated without (upper row) and with *P. simiae* WCS417r for two weeks.

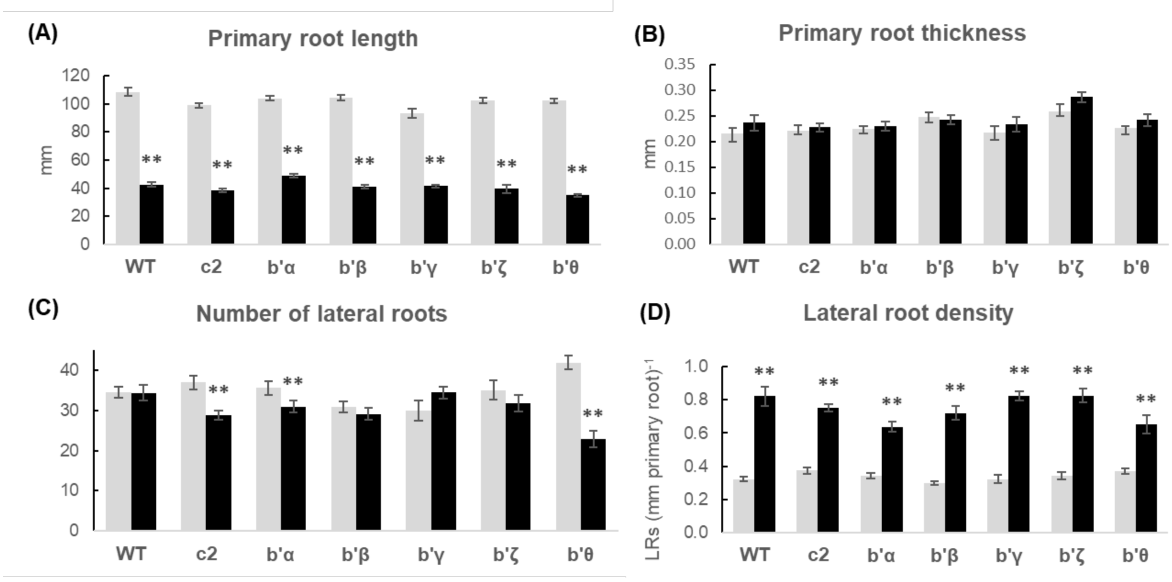

Figure S5. Growth parameters of WT, *c2* and various *b'*-mutants treated with *P. simiae* WCS417r. Seedlings of Arabidopsis WT and mutants (*c2, b'* alpha, beta, gamma, zeta, theta) were cultivated without (grey bars) and with (black bars) *P. simiae* WCS417r for two weeks. (A) primary root length; (B) primary root thickness; (C) number of lateral roots; and (D) lateral root density. Data are means ± SE of 15 plants (n=15). According to student's t-test and p-value < 0.05, columns marked with two asterisks are significantly different from the control without bacteria.
